# Modeling the distributions of tegu lizards in native and potential invasive ranges

**DOI:** 10.1038/s41598-018-28468-w

**Published:** 2018-07-05

**Authors:** Catherine S. Jarnevich, Mark A. Hayes, Lee A. Fitzgerald, Amy A. Yackel Adams, Bryan G. Falk, Michelle A. M. Collier, Lea’ R. Bonewell, Page E. Klug, Sergio Naretto, Robert N. Reed

**Affiliations:** 1 U.S. Geological Survey, Fort Collins Science Center, 2150 Centre Ave Bldg C, Fort Collins, CO 80526 USA; 2 Cherokee Nation Technologies, Fort Collins Science Center, 2150 Centre Ave Bldg C, Fort Collins, CO 80526 USA; 3 Biodiversity Research and Teaching Collections, Department of Wildlife and Fisheries Sciences, Texas A & M University, College Station, Texas USA; 4 U.S. Geological Survey, Everglades National Park, Homestead, Florida USA; 5 Laboratorio de Biología el Comportamiento. IDEA, Instituto de Diversidad y Ecología Animal (CONICET y Universidad Nacional de Córdoba), Av. Vélez Sarsfield 299, Córdoba, Argentina; 6 Present Address: Normandeau Associates, Inc., 4581 NW 6th Street, Suite A, Gainesville, FL 32609 USA; 7 Present Address: National Park Service, Everglades National Park, Everglades National Park, 40001 SR 9336, Homestead, FL 33034 USA; 8 Present Address: U.S. Department of Agriculture APHIS, Wildlife Services, National Wildlife Research Center, North Dakota Field Station, NDSU Biological Sciences Dept. 2715, P.O. Box 6050, Fargo, ND 58108 USA

## Abstract

Invasive reptilian predators can have substantial impacts on native species and ecosystems. Tegu lizards are widely distributed in South America east of the Andes, and are popular in the international live animal trade. Two species are established in Florida (U.S.A.) - *Salvator merianae* (Argentine black and white tegu) and *Tupinambis teguixin sensu lato* (gold tegu) – and a third has been recorded there— *S. rufescens* (red tegu). We built species distribution models (SDMs) using 5 approaches (logistic regression, multivariate adaptive regression splines, boosted regression trees, random forest, and maximum entropy) based on data from the native ranges. We then projected these models to North America to develop hypotheses for potential tegu distributions. Our results suggest that much of the southern United States and northern México probably contains suitable habitat for one or more of these tegu species. *Salvator rufescens* had higher habitat suitability in semi-arid areas, whereas *S. merianae* and *T. teguixin* had higher habitat suitability in more mesic areas. We propose that Florida is not the only state where these taxa could become established, and that early detection and rapid response programs targeting tegu lizards in potentially suitable habitat elsewhere in North America could help prevent establishment and abate negative impacts on native ecosystems.

## Introduction

The impacts of invasive predators on native biodiversity are increasingly acute and recognized as principal drivers of biodiversity loss^1^. Invasive species cause extinctions and irreversible ecosystem-wide changes in biodiversity, alter community-level processes, and transport pathogens, any of which can result in negative effects on ecosystem functions and services^2^. Invasive reptile species cause substantial harm to native populations and ecosystems^3–5^. Characteristics of reptiles, including low energetic requirements and high reproductive potential, contribute to their potential as efficient invaders that are difficult to eradicate^3,4^. Burgeoning populations of invasive reptiles can have substantial impacts on native species and ecosystems, including endangered species and ecologically and economically important species (e.g.^6–8^).

Tegus (Squamata: Teiidae; 3 species in *Salvator*, 7 in *Tupinambis*)^9^ are terrestrial lizards widely distributed in South America east of the Andes^10,11^. Individuals of at least three species of tegus have been documented outside of their native ranges. *Salvator merianae* (Argentine black and white tegu) and *Tupinambis teguixin sensu lato* (gold tegu) are established in Florida, while *S. rufescens* (red tegu) has also been documented in Florida but is not known to be established^12,13^. Elsewhere, *T. teguixin* and *S. merianae* have respectively invaded the islands San Andres, Colombia^14^ and Fernando de Noronha, Brazil^15^. Reptiles are the second most abundant taxon in the international pet trade^16^, and while tegus are increasingly recognized as potentially invasive species they have remained common in international live animal trade. Between 2000 and 2015, importers in the U.S. reported gross imports of over 79,000 live tegus of the genera *Salvator* and *Tupinambis* according to the CITES trade database (CITES trade statistics derived from the CITES Trade Database, UNEP World Conservation Monitoring Centre, Cambridge, UK). An unknown additional number of tegus are produced in the U.S. via captive breeding. Cumulatively, trade in live tegus in the U.S. is large and ownership of tegus is usually not restricted by states or municipalities, suggesting potentially high propagule pressure that could promote the establishment of additional extralimital populations. *Salvator merianae* populations appear to be expanding in Florida, and ongoing programs are aimed at understanding and confronting the conservation challenges of eradicating and controlling *S. merianae*^17^.

Tegus exhibit a number of life-history characteristics that may predispose them to be successful invaders. They are predacious omnivores with relatively rapid maturation, high reproductive output, large body size, and a relatively long lifespan^18,19^. Tegus are habitat and dietary generalists that live in various disturbed and undisturbed forest types and around urban areas and human settlements^10,20^. *Salvator merianae* and *S. rufescens* reach latitudes with relatively cold winter temperatures, coping with cold temperatures or drought by entering into prolonged dormancy or shorter bouts of torpor^21^. Tegus are opportunistic feeders, consuming a surprisingly diverse variety of foods including plant matter, fruit, insects, mollusks, every class of vertebrates, and carrion (e.g.^22^). In Argentina, Paraguay, and Bolivia, *S. merianae* and *S. rufescens* have proven resilient to intensive market hunting^18^, suggesting that eradication of invasive populations would be challenging. Despite an average of 1.9 million skins per year in world trade throughout the 1980s, with >3 million skins entering trade in peak years, there are no known localities where tegus have been extirpated as a result of hunting^23,24^.

Our first goal was to model and map the native-range distributions of the 3 large-bodied tegu species that have been documented in Florida. Several other species of tegus were not considered for this analysis because they are not known to have invaded North America and their distributions and habitat affinities are not well known. We used a suite of five species distribution models (SDMs) based on presumed biologically meaningful input variables to create maps of potentially suitable habitat for these species in South America. The second goal was to project these native range models of potentially suitable habitat for tegus into North America, thus providing the ability to assess relative invasion risk for each species and for tegus overall. Species distribution models are a commonly used tool to identify suitable habitat for potential biological invaders^25,26^. We also developed a distribution model based on occurrences for all 3 species combined. This would be informative because we do not know whether the distributional boundaries between the species in South America are due to purely physiological responses to environmental conditions, or are based on historical biogeography and/or biotic interactions (such as competition). If tegu distributions were due to biotic interactions among tegu species, for example, we would not capture the full potential range of a single species within North America when its congeners are not in the available species pool. The species distribution models and maps generated in this analysis are empirically-derived geospatial hypotheses of distributions of these tegu species in South America and potentially suitable habitat in North America.

## Results

**Figure 1:**
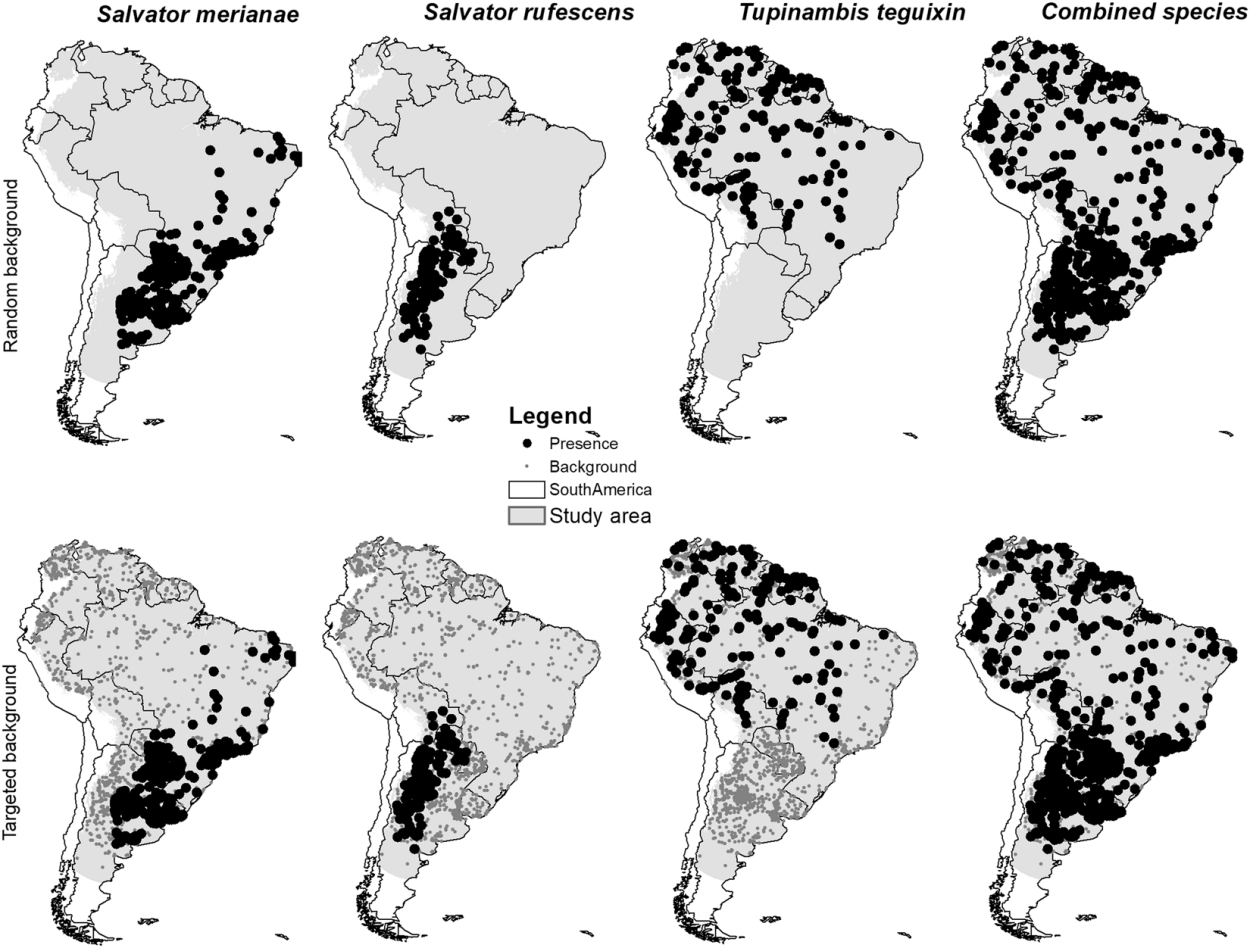
Spatial extent and occurrence records (black dots) for *Salvator merianae* (Argentine black and white tegu), *Salvator rufescens* (red tegu), *Tupinambis teguixin* (gold tegu), and these records combined in South America. The study area for analysis is shown in gray. Maps use the geographic coordinate system and were built using Esri ArcGIS 10.5 (www.esri.com/sofware/arcgis).

**Figure 2:**
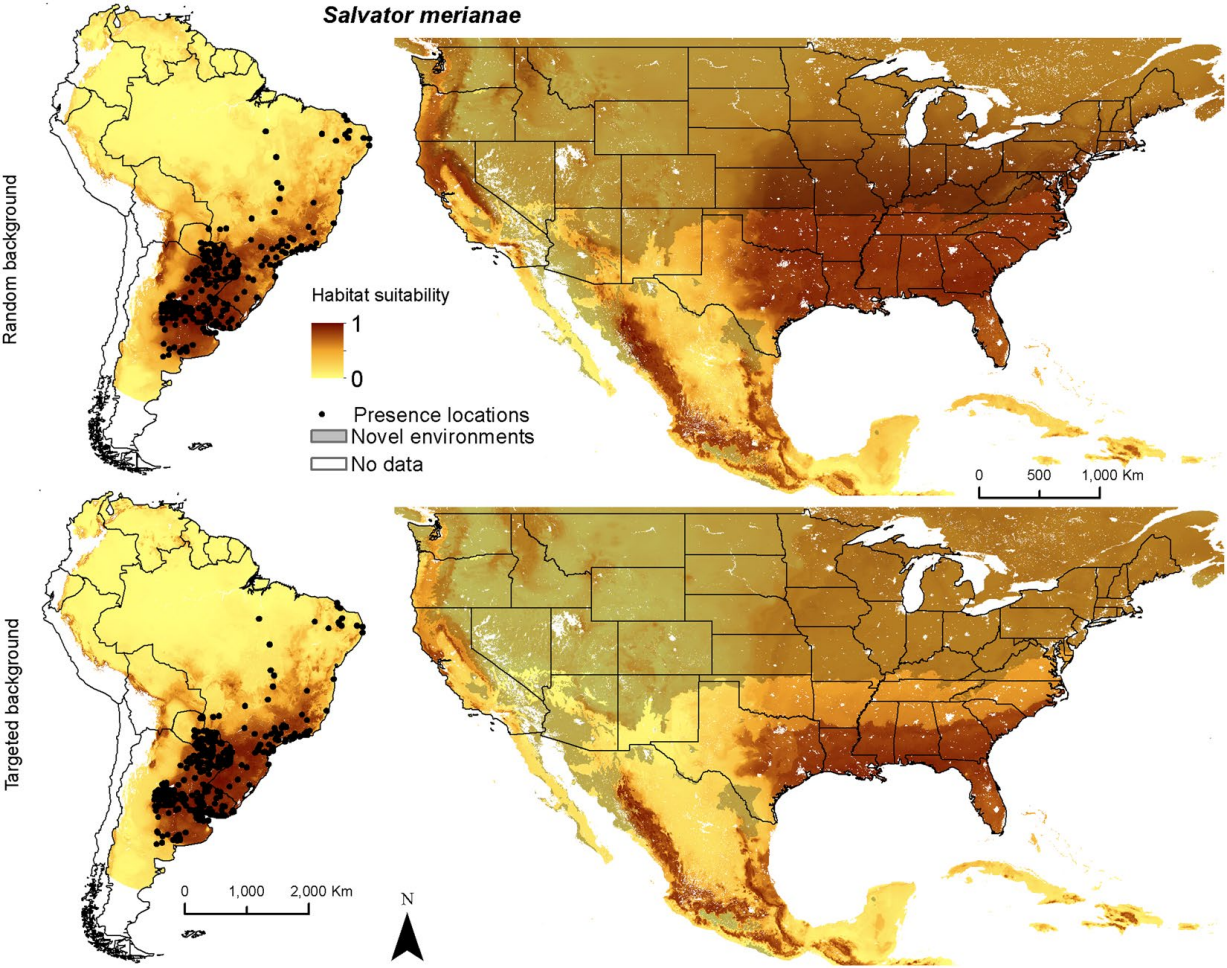
Ensemble models of average habitat suitability from 5 model algorithms including a generalized linear model, multivariate adaptive regression splines, boosted regression trees, random forest, and Maxent for *Salvator merianae* (Argentine black and white tegu) trained on data in South America and applied to North America (columns). Models were produced with random and targeted background data (rows). Areas identified as having novel environmental conditions based on the Multivariate Environmental Similarity Surface are shown with a transparent gray layer. Maps use the geographic coordinate system and were built using Esri ArcGIS 10.5 (www.esri.com/sofware/arcgis).

**Figure 3:**
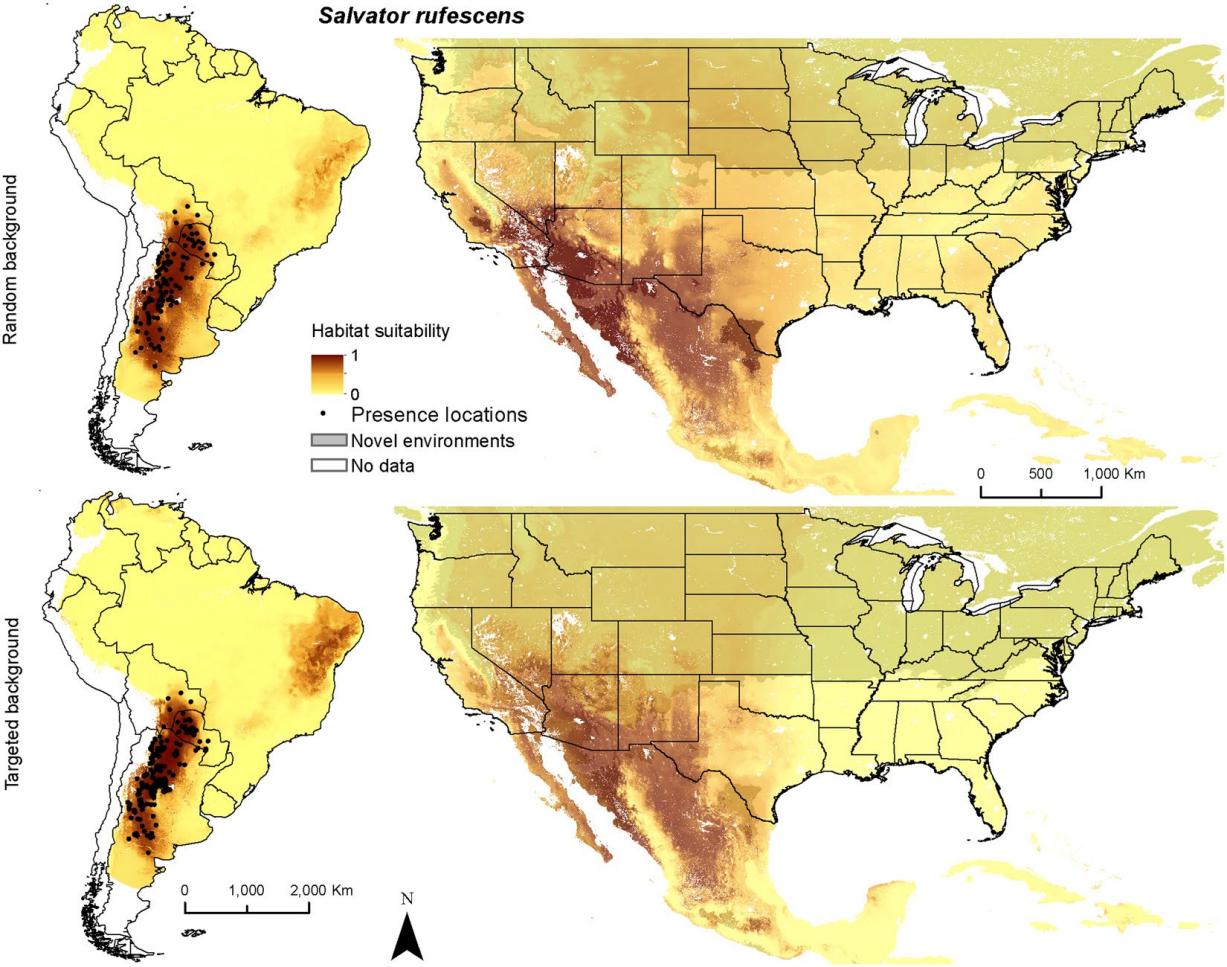
Ensemble models of average habitat suitability from 5 model algorithms including a generalized linear model, multivariate adaptive regression splines, boosted regression trees, random forest, and Maxent for *Salvator rufescens* (red tegu) trained on data in South America and applied to North America (columns). Models were produced with random and targeted background data (rows). Areas identified as having novel environmental conditions based on the Multivariate Environmental Similarity Surface are shown with a transparent gray layer. Maps use the geographic coordinate system and were built using Esri ArcGIS 10.5 (www.esri.com/sofware/arcgis).

**Figure 4:**
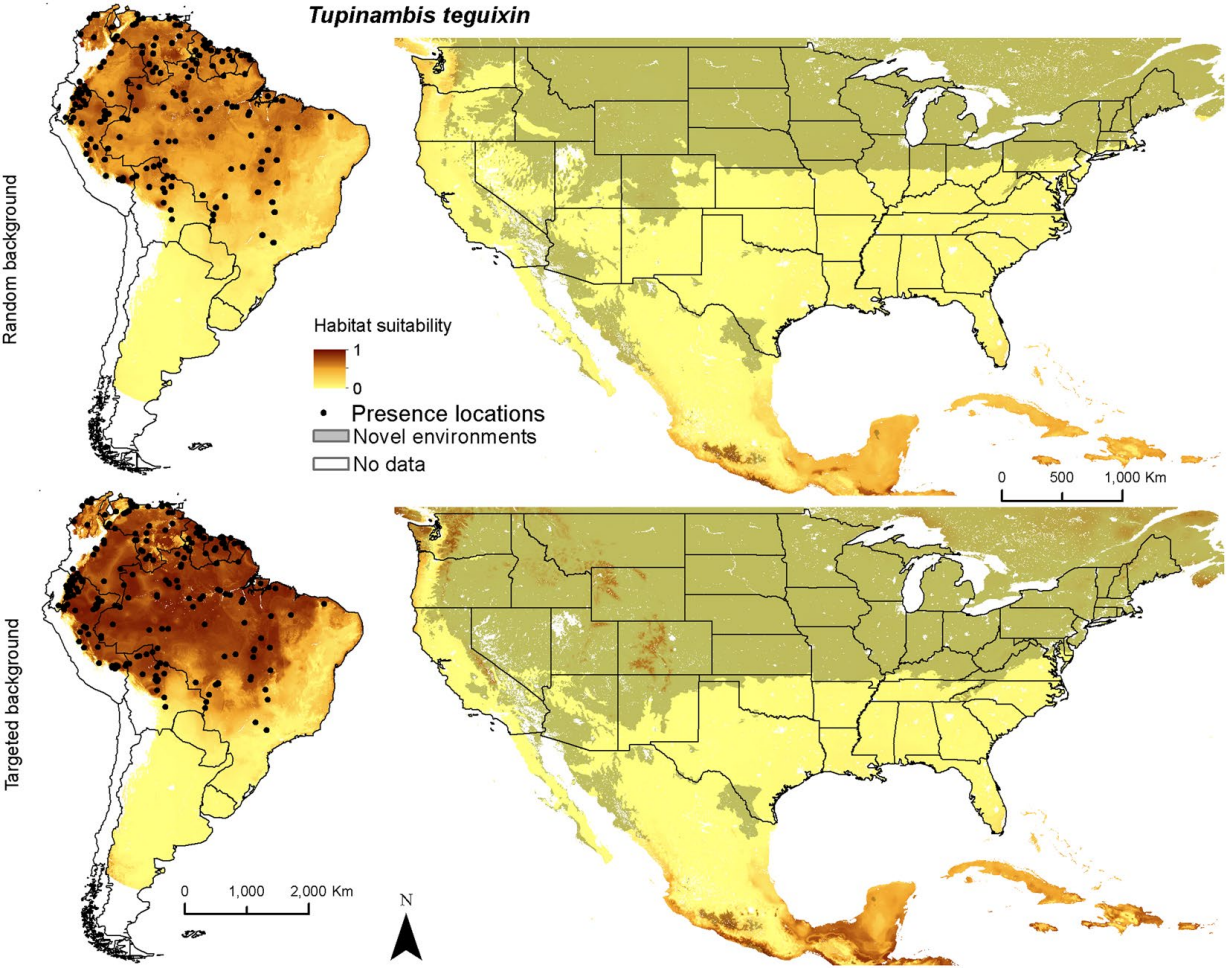
Ensemble models of average habitat suitability from 5 model algorithms including a generalized linear model, multivariate adaptive regression splines, boosted regression trees, random forest, and Maxent for *Tupinambis teguixin* (gold tegus) trained on data in South America and applied to North America (columns). Models were produced with random and targeted background data (rows). Areas identified as having novel environmental conditions based on the Multivariate Environmental Similarity Surface are shown with a transparent gray layer. Maps use the geographic coordinate system and were built using Esri ArcGIS 10.5 (www.esri.com/sofware/arcgis).

**Figure 5:**
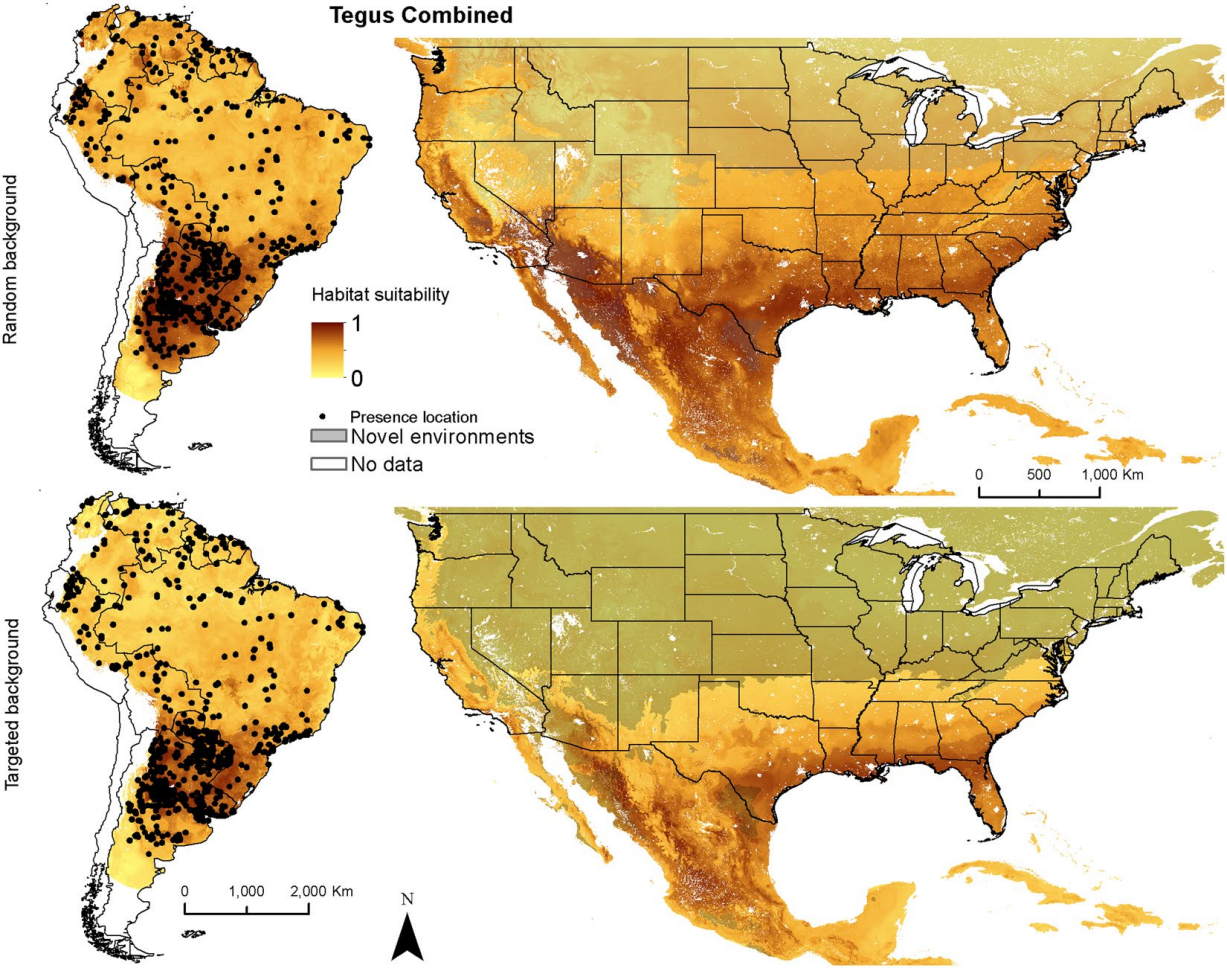
Ensemble models of average habitat suitability from 5 model algorithms including a generalized linear model, multivariate adaptive regression splines, boosted regression trees, random forest, and Maxent for the 3 species of tegus combined trained on data in South America and applied to North America (columns). Models were produced with random and targeted background data (rows). Areas identified as having novel environmental conditions based on the Multivariate Environmental Similarity Surface are shown with a transparent gray layer. Maps use the geographic coordinate system and were built using Esri ArcGIS 10.5 (www.esri.com/sofware/arcgis).

We fit species distribution models using 194 thinned (subset of records ≥50 km apart) and 372 un-thinned locations for *Salvator merianae*, 97 thinned and 245 un-thinned locations for *S. rufescens*, and 159 thinned and 199 un-thinned *Tupinambis teguixin* locations. The combined map was based on 448 thinned and 807 un-thinned locations for tegu (Fig. 1). Training split area under the receiver operating characteristic curve (AUC) and cross-validation AUC scores ranged from 0.67–0.97, with little variation in AUC scores among model algorithms for each species or the species combined models (Table 1). All models performed well with AUC values > 0.7^27^ except for combined models with random background. Sensitivity ranged from 0.28–0.92 (Table 1), with more variation for *T. teguixin* and combined models. Specificity values had a similar pattern. Percent correctly classified values were again similar except for RF for *T. teguixin* and combined models. True skill statistic also had good performance, with almost all values > 0.4 except all combined models and all random and RF targeted *T. teguixin* models. The top performing model algorithm varied by SDM metric, so no model algorithm was consistently superior. Thus, we combined all models for each species to create ensemble maps (Figs 2–5). Given the performance of the models and the general difference of 0.05 or less between AUC-train and AUC-CV (cross-validation mean) values, we felt that model complexity and fit were well balanced.

**Table 1:** Species distribution model evaluation metrics for *Salvator merianae* (Argentine black and white tegu), *Salvator rufescens* (red tegu), *Tupinambis teguixin* (gold tegus), and the 3 tegu species combined in South America, using 5 model algorithms (rows) and two different background approaches (shown as random/targeted).

SDM	AUC-train	AUC-CV	Sensitivity	Specificity	PCC	TSS	
*S. merianae*							
GLM	0.87/0.83	0.85/0.82	0.83/0.74	0.79/0.75	79.13/74.67	0.62/0.49	
MARS	0.87/0.89	0.86/0.88	0.85/0.79	0.76/0.82	76.21/81.16	0.61/0.61	
BRT	0.92/0.94	0.87/0.91	0.85/0.80	0.77/0.84	77.18/83.37	0.62/0.64	
RF	0.88/0.92	0.88/0.92	0.71/0.74	0.85/0.91	85.16/87.11	0.56/0.65	
Maxent	0.89/0.91	0.87/0.90	0.80/0.82	0.81/0.84	80.51/83.15	0.61/0.65	
*S. rufescens*							
GLM	0.95/0.93	0.94/0.94	0.89/0.87	0.87/0.88	87.21/87.85	0.76/0.75	
MARS	0.94/0.94	0.93/0.94	0.92/0.87	0.84/0.88	84.07/88.23	0.76/0.76	
BRT	0.95/0.97	0.94/0.95	0.92/0.84	0.87/0.91	87.14/89.90	0.79/0.75	
RF	0.94/0.96	0.94/0.96	0.86/0.77	0.90/0.94	90.10/91.73	0.76/0.71	
Maxent	0.94/0.96	0.94/0.95	0.88/0.87	0.87/0.90	87.23/89.95	0.75/0.78	
*T. teguixin*							
GLM	0.74/0.83	0.73/0.81	0.73/0.72	0.58/0.75	58.11/74.65	0.31/0.47	
MARS	0.75/0.85	0.73/0.83	0.77/0.71	0.59/0.76	58.93/75.74	0.36/0.47	
BRT	0.82/0.89	0.74/0.84	0.53/0.69	0.72/0.80	71.25/78.43	0.25/0.49	
RF	0.77/0.86	0.77/0.85	0.28/0.40	0.93/0.89	91.52/83.94	0.20/0.30	
Maxent	0.82/0.88	0.77/0.85	0.68/0.73	0.68/0.78	68.22/77.53	0.36/0.51	
Combined tegus							
GLM	0.69/0.73	0.67/0.73	0.59/0.69	0.72/0.69	71.16/69.42	0.31/0.39	
MARS	0.70/0.76	0.67/0.75	0.58/0.69	0.69/0.69	68.48/69.19	0.27/0.38	
BRT	0.82/0.80	0.70/0.75	0.62/0.66	0.68/0.69	68.05/67.42	0.30/0.35	
RF	0.71/0.76	0.72/0.76	0.39/0.63	0.87/0.759	85.03/69.72	0.26/0.38	
Maxent	0.73/0.77	0.70/0.75	0.57/0.70	0.75/0.69	74.46/69.46	0.32/0.39	

Abbreviations: SDM, species distribution model; AUC, area under the curve; AUC-train, training split AUC; AUC-CV, Cross-validation mean AUC; PCC, percent correctly classified; TSS, True skills statistic; GLM, generalized linear model (logistic regression); MARS, multivariate adaptive regression splines; BRT, boosted regression trees; RF, random forest.

For *S. merianae*, mean temperature of the coldest quarter was most important, followed by annual precipitation except for targeted GLM where it was dropped (Table 2). All SDM approaches suggested that habitat suitability improved as the mean temperature of the coldest quarter increased above 5 °C, with all approaches predicting maximum habitat suitability with mean temperature of the coldest quarter of approximately 5–15 °C (Supporting Information). All approaches suggested that habitat suitability improved in areas with annual precipitation >1,000 mm. The native range ensemble distribution maps (Fig. 2) predicted suitable habitat for *S. merianae* in the subtropical and humid ecoregions of southeastern South America.

**Table 2:** Variable importance using change in Area Under the Curve (AUC) for each environmental layer using 5 species distribution modelling algorithms ^a^(columns) by two different background approaches (shown as random/targeted) to model the distributions of *Salvator merianae* (Argentine black and white tegu), *Salvator rufescens* (red tegu), *Tupinambis teguixin* (gold tegus), and the 3 combined in South America. The most important predictor in each is in bold.

	GLM	MARS	BRT	RF	Maxent	Mean ΔAUC
*S. merianae*						
Warmest month (BIO5)	0.009/na	0.002/0.006	na/na	0.016/0.023	0.007/0.006	0.009/0.012
Coldest quarter (BIO11)	**0.289**/**0.335**	**0.247**/**0.258**	**0.256**/**0.276**	**0.269**/**0.311**	**0.278**/**0.278**	**0.268**/**0.291**
Precipitation (BIO12)	0.097/na	0.092/0.153	0.072/0.197	0.047/0.098	0.08/0.162	0.078/0.153
PET	na/0.044	0.014/0.006	na/na	0.02/0.042	0.005/0.008	0.013/0.025
*S. rufescens*						
Warmest month (BIO5)	0.005/na	0.024/0.005	na/na	0.002/0.009	0.02/0.004	0.013/0.006
Coldest quarter (BIO11)	0.121/na	0.119/0.119	0.084/na	0.08/0.084	**0.134**/0.075	0.101/0.093
Precipitation (BIO12)	**0.197**/**0.3**	**0.276**/**0.303**	**0.122**/**0.191**	**0.085**/**0.163**	0.127/**0.142**	**0.161**/**0.22**
PET	0.071/0.097	0.034/0.045	na/0.068	0.035/0.054	0.023/0.07	0.041/0.067
*T. teguixin*						
Warmest month (BIO5)	0.042/na	0.017/0	na/na	0.107/0.014	0.026/0	0.048/0.003
Coldest quarter (BIO11)	**0.232**/**0.188**	**0.212**/**0.283**	**0.168**/**0.203**	**0.266**/**0.208**	**0.172**/**0.241**	**0.210**/**0.225**
Precipitation (BIO12)	0.010/0.049	0.02/0.028	0.053/0.043	0.139/0.049	0.057/0.044	0.056/0.043
PET	na/na	0.006/0.001	na/na	0.124/0.004	0.05/0.004	0.06/0.003
Combined tegus						
Warmest month (BIO5)	0.064/0.058	0.016/0.013	na/na	0.048/0.06	0.022/0.025	0.038/0.039
Coldest quarter (BIO11)	**0.146**/**0.252**	**0.143**/**0.228**	**0.154**/**0.213**	**0.133**/**0.244**	**0.125**/**0.231**	**0.14**/**0.234**
Precipitation (BIO12)	0.048/0.015	0.012/0.048	0.035/0.033	0.047/0.05	0.016/0.041	0.032/0.038
PET	0.014/na	0.014/0.048	0.035/na	0.052/0.03	0.021/0.02	0.027/0.032

^a^Abbreviations: SDM, species distribution model; AUC, area under the curve; ΔAUC, change in AUC; GLM, generalized linear model (logistic regression); MARS, multivariate adaptive regression splines; BRT, boosted regression trees; RF, random forest; PET, potential evapotranspiration; na, not applicable.

For *S. rufescens*, annual precipitation was most important for all models but random Maxent, generally followed by mean temperature of the coldest quarter (Table 2). All SDM approaches suggested that habitat suitability improved as the mean temperature of the coldest quarter increased above 5 °C, with all approaches predicting maximum habitat suitability with mean temperature of the coldest quarter of approximately 5–20 °C (Supplementary Fig. S1). All approaches suggested that habitat suitability improved in areas with annual precipitation <1,500 mm (Supplementary Fig. S1). The ensemble distribution maps for *S. rufescens*’ native range (Fig. 3) included the arid Chacoan region of southcentral South America, where it is endemic.

For *T. teguixin*, the mean temperature of the coldest quarter had the highest mean increase in AUC, followed by annual precipitation, maximum temperature of the warmest month, and potential evapotranspiration (Table 2). All SDM approaches suggested that habitat suitability improved as the mean temperature of the coldest quarter increased above 15 °C, with all approaches predicting maximum habitat suitability with increasing mean temperature of the coldest quarter (Supplementary Fig. S1). All approaches suggested that habitat suitability improved in areas with annual precipitation >1,000 mm (Supplementary Fig. S1). The native range ensemble distribution maps (Fig. 4) suggest that the distribution of *T. teguixin* is associated with tropical ecoregions of central and northern South America.

For combined-species models, mean temperature of the coldest quarter had the highest mean increase in AUC, followed by annual precipitation, maximum temperature of the warmest month, and potential evapotranspiration (Table 2). All SDM approaches predicted maximum habitat suitability with mean temperature of the coldest quarter of 10–15 °C (Supplementary Fig. S1). All approaches suggested that habitat suitability improved in areas with higher annual precipitation. The native range ensemble distribution maps (Fig. 5) suggested different distributions based on the background method.

Both North American ensemble maps for *S. merianae* predict potentially high habitat suitability throughout the southeastern United States (Fig. 2). Random background suggests higher suitability in the mid-Atlantic region, Great Plains, and Pacific coast than does targeted background. In México relatively higher habitat suitability is predicted on the flanks of the Sierra Madre Occidental and the Sierra Madre Oriental. Both ensemble maps for *S. rufescens* show relatively higher habitat suitability throughout arid and semi-arid regions of the southwestern United States, including the Southwestern Tablelands, and portions of the Mojave, Chihuahua, and Sonoran deserts (Fig. 3). Random background predicts relatively higher suitability in the southern Texas Plains and farther north. In México higher habitat suitability is predicted in the arid regions of northern México and the Baja California desert. Both ensemble maps for *T. teguixin* suggest that relatively higher habitat suitability may be restricted to tropical regions in southern México and the Caribbean, especially in areas dominated by tropical forests (Fig. 4). Finally, both combined tegu ensemble maps suggest that habitat may be relatively suitable for at least one species of large-bodied tegus throughout much of the southern United States and most of México. The random background models included higher suitability farther north across the United States and in the United States portion of the Sonoran desert. The MESS maps for these species in North America depict large areas of the northern United States with environmental conditions outside those in the training data; thus, we have less confidence in our predictions of habitat suitability for tegus in these areas.

## Discussion

To our knowledge this is the first attempt to statistically model and map the potential distributions of tegu species in the Americas. Li *et al*.^28^ included some data for two species of tegus along with >150 other herptiles in global analyses of susceptibility of ecoregions to herpetofaunal invasions under future climate scenarios. While they did not focus on individual species’ habitat suitability per se, their results pointed to the potential for invasion of tegus and many other established non-native species into other ecoregions. Invasive species may also undergo niche shifts in invaded ranges^29^, particularly for species with small native ranges^30^. For this reason, some studies have used an iterative process to develop models for established invasive species as they spread to potentially novel environments^31^ or developed models with data from both native and invaded ranges to capture the full range of environments occupied by the species^32^. The tegu lizard species that are established in the USA have a very large geographic native range and use a variety of landscapes throughout their native ranges and our models predict large areas in North America may be suitable for their establishment. Under climate change scenarios in North America and the potential for niche shifts to occur, the invasion of tegu lizards could possibly be even more extensive than our models indicate but correlative models may not be appropriate to predict these types of changes^33^. Our results support the conjecture that these large predacious lizards have the potential to occupy extensive areas and most ecoregions in the southern portion of North America, where they may exert negative impacts on native fauna.

Mean annual precipitation and mean temperature of the coldest quarter were the environmental layers with most support among all models. In their native range, *S. merianae* is known to be associated with the humid pampas, humid Chaco, savannahs, moist forests, and Espinal of Argentina, eastern Paraguay, Uruguay, and southeastern Brazil^11^. *Salvator rufescens* occurs in dry Chaco, Monte, and Espinal of Argentina, western Paraguay, and southeastern Bolivia^34–36^. *Tupinambis teguixin* records were associated with the moist forests and savannahs of northern South America. In particular, *S. merianae* and *S. rufescens* are known from areas with relatively colder winter temperatures. *Salvator rufescens* occurs in semi-arid regions, mainly the dry Chaco, while *S. merianae* occurrences range over a very broad area with moderate precipitation. Although *S. merianae* and *S. rufescens* are sympatric in wet-to-dry transition zones in the Chaco^34–36^, the bulk of the range of *S. rufescens* lies in more arid regions. Our analyses revealed patterns of occurrence of *S. rufescens* in areas with colder winter temperatures than *S. merianae* and *T. teguixin*. In general, our findings corroborate those of Lanfri *et al*.^37^, who also concluded that precipitation and temperature variables were the strongest predictors of the distributions of *Salvator* species in South America. The pattern of these distributions in the native ranges, together with our model results suggest that cold winter temperatures and annual precipitation are likely to strongly influence tegu distributions in South America.

The invasion of *S. merianae* is increasingly well documented in Florida and substantial concern has been expressed about the potential impacts of this species on native species and ecosystems (e.g.^17,38^). As of March 2017, >3,200 observations of tegus, mostly *S. merianae*, were reported in the wild in Florida^39^; many of these records came from animals that were removed from southern Florida habitats via live-trapping. The time series of expanding occurrence records and knowledge of the species’ movements, seasonal activity, and reproduction^40^ demonstrate significant expansion of these populations over time. Modeled *S. merianae* habitat suitability suggests that populations may be able to expand into northern Florida as well as the coastal and southeastern plains and Piedmont of the United States. We are not aware of any major barriers to the expansion of *S. merianae* in this vast region.

Our results predicted less suitable habitat for *S. rufescens* and *T. teguixin* in Florida than in other parts of North America. Our models and maps suggest that large regions of suitable habitat for *S. rufescens* exist in the semiarid southwestern United States; there may be more and better habitat for this species compared to what is potentially available for *S. rufescens* in the southeastern United States. The North American map (Fig. 3) also suggests areas that could be monitored for presence of *S. rufescens* and help inform policies and/or management actions aimed at keeping *S. rufescens* from establishing in the American Southwest.

In this analysis, we adopted a risk analysis perspective that uses known occurrence records with background locations to develop species distribution models and maps, while keeping in mind the key caveats associated with the use of background data and the maps derived from them^41,42^. The approach used here may be useful for assessing invasion risk of many ectotherms, especially non-native reptiles. Because of the uncertainty introduced by background and aggregated presence data, we emphasize that our models could be improved in the future by including presence-absence data derived from well-designed and spatially-balanced sampling schemes (e.g.^43^). Although such sampling has not yet been completed for tegus in South America, the limits of the species’ ranges appear to be well known^35,37,44^. Although it is not possible to completely correct for the sampling bias inherent in Global Biodiversity Information Facility (GBIF) and all other presence-only sources when conducting habitat suitability modeling, we implemented two methods that have been proposed to handle bias in occurrence data. Results of implementing these methods exhibited commonalities, giving us more confidence in the robustness of the analysis. Habitat suitability values are not directly comparable because each background and species relied on different input data, but the relative patterns are comparable, and thus the commonalities in patterns could provide guidance for management decisions.

Our SDMs did not include all possible parameters that could affect habitat suitability for tegus in North America. For example, we do not know how interactions between tegus and predators or competitors may help shape the species’ distributions, or how availability of food resources in North America may influence tegu abundance or potential for population establishment. We are also currently unable to predict the capacity for acclimation of tegus in new environments, but they appear to be adaptable generalists that exhibit substantial variation in foraging mode, nesting habits, and climatic regimes. The North America models represent locations with environments similar to those occupied by tegus in South America, but if the selected environmental layers do not constrain habitat, then tegus may be capable of invading areas in North America that were not considered highly suitable in this analysis.

These models and maps of habitat suitability, combined with knowledge of the species’ natural history, suggests that these large omnivorous lizards might find suitable habitat over broad swaths of North America. Tegus could therefore impact native flora and fauna in the southeastern United States, elsewhere in the United States, its territories and México, and plausibly in Central America (Figs 2–5, Supplementary Fig. S2). Given their omnivorous diet^45^, the reproductive success of terrestrial egg-laying species could be negatively impacted^38,46^ along with economic losses to agricultural industries^47^. Tegus are thought to be important seed dispersers in the Neotropics and therefore may alter structure and function of invaded ecosystems^48^. Our results also serve as a basis for developing testable predictions about the limits of dispersal and suitability of habitat for tegus in North America. In addition to careful documentation of the ecological impacts of tegus on native terrestrial fauna, future studies could focus on patterns of dispersal in tegus and how to constrain the spread of these species in novel environments. This work is an important first step in understanding the continental-scale conservation challenges presented by non-native tegus in North America.

## Methods

We compiled occurrence records for *S. merianae*, *S. rufescens*, and *T. teguixin* in South America from the GBIF (www.gbif.org), the HerpNET data portal (www.herpnet.org), publications^35,37,44^, and other credible records from herpetologists known to the authors. *Tupinambis* comprises a species complex of 4 or more species^9^. Pending molecular and/or morphological identification of the species currently present in southern Florida, and because species limits and distributions of this complex in the native range are poorly understood, we retained the long-held taxonomy of *T. teguixin* as one taxonomic entity. We did not include locations from established populations in the United States for the two established species because they have not been present long and their introduced range covers a relatively small geographic area. The range of conditions in which they will eventually occur is not understood, and including records from these recent invaders would likely violate the assumption in SDMs that geographic distributions are at equilibrium.

We eliminated occurrence records identified only to state, province, or country, or which had an identifiable spatial error of >50 km. We compared our occurrence maps to those in key publications (e.g.^35,44,49^), and excluded locality records that could not be verified. We further restricted data based on geography, eliminating areas >2,500 m elevation (above the highest documented elevation known for these tegu lizard species) and the southernmost portion of South America beyond the known southernmost records for any tegu. Our analyses were conducted with a cell resolution of 30 arc-seconds (~1 km^2^) in the South American Albers Equal Area Conic projection (NAD83 datum).

To develop the modeling strategy and identify potential environmental layers, we convened a 3-day workshop attended by AYA, BGF, CSJ, LAF, LRB, MAH, MAC, PEK, and RNR. We based our selection of potential environmental layers (see Table 3) on a body of research that supports the influence of biophysical factors on the distributions and behavior of ectotherms (e.g.^50^). To arrive at the final set of environmental layers (Table 3), we conducted a covariate correlation analysis and eliminated one variable from each pair with r > |0.70| (using maximum of Pearson, Spearman, and/or Kendall correlation coefficients), retaining the hypothesized most biologically relevant variable^51^.

**Table 3:** Environmental layers considered and used in modeling patterns of South American distribution of *Salvator merianae* (Argentine black and white tegu), *Salvator rufescens* (red tegu), *Tupinambis teguixin* (gold tegus), and these species combined. All data were 30 arc-second resolution.

Variable	Units	Justification	Source
Annual mean temperature (BIO1)	°C	Distribution limits of squamates are constrained by low temperatures at continental scales^60^	www.worldclim.org (average of values from 1950–2000)^61^
Maximum temperature of the warmest month (BIO5)*	°C	Although behavioral thermoregulation allows some avoidance of thermal stress, thermal tolerances are conserved in lizard lineages and influence habitat suitability^62–64^	www.worldclim.org (average of values from 1950–2000)^61^
Minimum temperature of the coldest month (BIO6)	°C	As per previous justification	www.worldclim.org (average of values from 1950–2000)^61^
Mean temperature of the warmest quarter (BIO10)	°C	Temperatures need to be warm enough to permit at least a 7-month activity season, corresponding to seasonal activity period in South America^49^ and observed activity of a few individuals in Florida^40^	www.worldclim.org (average of values from 1950–2000)^61^
Mean temperature of the coldest quarter (BIO11)*	°C	Moderate winter temperatures would permit at least a 7-month activity season, corresponding to seasonal activity period in South America^49^ and observed activity of a few individuals in Florida^40^	www.worldclim.org (average of values from 1950–2000)^61^
Annual precipitation (BIO12)*	mm	Influences ecosystem dynamics and primary productivity	www.worldclim.org (average of values from 1950–2000)^61^
Moderate Resolution Imaging Spectroradiometer [MODIS] Phenology EVI length of season	Days	Season length should influence resource availability, which in turn influences fitness and survival in many animals including lizards^65^	www.modis.gsfc.nasa.gov ^66^
Mean annual potential evapotranspiration (PET)*	mm	Measures amount of water evaporated and transpired if water is not limiting; interacts to influence major vegetation associations at continental scales and is a strong predictor of animal species richness world-wide^67^	www.nysg.umt.edu/project/mod16 (mean of 2000 to 2009 MOD16 A3 PET dataset)
Solar radiation index (SRI)	No units	Important for ectothermic species to meet their thermoregulatory needs	Keating *et al*.^68^; equation 2, pp. 1345, using latitude, slope, and aspect derived from USGS map layers

^*^Variables used in the final set of environmental layers.

We addressed sample bias and spatial autocorrelation in two ways. First, we examined geographic distance and spatially-structured variance among occurrence locations. We used the ‘spThin’ package for R to find the set of occurrence locations maximizing the number of records ≥50 km apart, which we considered likely to be spatially independent^52,53^. We used a random sample of 10,000 pixels from all available cells in the study area with a uniform inclusion probability for all cells on the study area as background. The random background locations were selected for each species, but were effectively the same because we used the same study area and random seed for each. Second, we followed the target-group background approach as proposed by Phillips *et al*.^54^. We downloaded all GBIF records for the family Teiidae (10.15468/dl.5xbem6) that were flagged as being georeferenced with no spatial issues, which, when reduced to one record per pixel within the study area, resulted in 1,200 background locations (Fig. 1). We merged these with the location data we aggregated for all tegu species to develop the target background locations so that the background data included both locations from GBIF and the tegu species location data from other sources. We developed models with these targeted background locations and the un-thinned presence data, assuming the two data sets would have similar sampling biases. The first method (thinned + random background) we refer to as random and the second (un-thinned + targeted background) we refer to as targeted.

We analyzed and mapped the potential distributions of each tegu species individually (*S. merianae*, *S. rufescens*, *T. teguixin*), as well as for a combined dataset using the occurrence data for the 3 tegu species. We used 5 SDM algorithms to model and map these distributions: generalized linear models using logistic regression and maximum likelihood estimation (GLM); multivariate adaptive regression splines (MARS); boosted regression trees (BRT); random forests (RF); and maximum entropy (Maxent), using 10-fold cross-validation for each approach. We used the Software for Assisted Habitat Modeling SAHM, version 2.2.3^55^; package for VisTrails software to fit species distribution models and calculate SDM performance metrics. Each SDM model produced an estimate of relative habitat suitability for each cell in the study area, given the variables used, expressed as continuous values between 0 and 1. For GLM, we used a bidirectional stepwise procedure using Akaike’s Information Criterion (AIC), considering all interactions and squared terms. For MARS, we used Mars Degree (Friedman’s *μ*) = 1 and GVL penalty = 2.0. For BRT, we used bag fraction = 0.5 with other values at default values. For RF, SAHM uses the tuneRF function to minimize out of bag error. We used the Maxent software version 3.3.3k^56^; as implemented in SAHM. To visualize potential distributions, we generated ensemble maps using the average of the relative habitat suitability estimate of the 5 models for each of the individual species and the combined species.

To evaluate SDM performance among the 5 algorithms for each model, we used the following metrics: AUC (AUC-train and AUC-CV); sensitivity (the model’s ability to predict true presences); specificity (the model’s ability to predict background cells as absence); percent correctly classified (PCC; the percentage of all cells that were correctly classified); and true skills statistic (TSS)^57^. For metrics that required a threshold (sensitivity, specificity, PCC, and TSS), we used the threshold that maximized the average value of sensitivity and specificity^57^. Models with values of AUC > 0.7 and TSS > 0.0.4 are deemed acceptable^27,58^. We examined the appropriateness of model complexity by looking at both the difference between AUC-train and AUC-CV, which provides information on how sensitive the model is to the data being used to fit it, and visual assessment of response curve complexity. We evaluated variable importance for each model based on change in the AUC statistic (ΔAUC) when values for that environmental layer were permutated between presence and background, then ranked environmental layers by mean ΔAUC across the validation runs. We also calculated the multivariate environmental similarity surface (MESS^59^) to identify areas in North America that had environmental conditions outside those contained in the model training data from South America. MESS maps were created by comparing the range of values in the training data for each environmental layer to each location being predicted to, providing an indication of the similarity of environmental conditions at the prediction location to the conditions used to develop the model. We created maps highlighting areas with negative values, which occur when at least one environmental variable has values outside the range of the training data.

### Data availability

The datasets generated during and/or analyzed during the current study are available in the ScienceBase repository, 10.5066/P9JZZE4W.

## Electronic supplementary material

Supplementary information
