# Supplementary material for: Modeling the distributions of tegu lizards in native and potential invasive ranges
Source: Sci Rep. 2018 Jul 5;8:10193. doi: 10.1038/s41598-018-28468-w (PMC6033913; doi:10.1038/s41598-018-28468-w)

**Title:** Modeling the distributions of tegu lizards in native and potential invasive ranges

**Short page title:** Modeling distributions of tegu lizards

**Authors**:

Catherine S. Jarnevich^1^, Mark A. Hayes^2*^, Lee A. Fitzgerald^3^, Amy A. Yackel Adams^1^, Bryan G. Falk^4**^, Michelle A. M. Collier^4**^, Lea’ R. Bonewell^1^, Page E. Klug^1***^, Sergio Naretto^5^, and Robert N. Reed^1^

^1^U.S. Geological Survey, Fort Collins Science Center, 2150 Centre Ave Bldg C, Fort Collins, CO, 80526, U.S.A.; Corresponding author email: jarnevichc@usgs.gov

^2^Cherokee Nation Technologies, Fort Collins Science Center, 2150 Centre Ave Bldg C, Fort Collins, CO, 80526, U.S.A.

^3^Biodiversity Research and Teaching Collections, Department of Wildlife and Fisheries Sciences, Texas A & M University, College Station, Texas, U.S.A.

^4^U.S. Geological Survey, Everglades National Park, Homestead, Florida, U.S.A.

^5^Laboratorio de Biología el Comportamiento. IDEA, Instituto de Diversidad y Ecología Animal (CONICET y Universidad Nacional de Córdoba), Av. Vélez Sarsfield 299. Córdoba, ARGENTINA

Current addresses:

* Normandeau Associates, Inc., 4581 NW 6^th^ Street, Suite A, Gainesville, FL 32609

**National Park Service, Everglades National Park, Everglades National Park, 40001 SR 9336, Homestead, FL 33034, U.S.A.

***U.S. Department of Agriculture APHIS, Wildlife Services, National Wildlife Research Center, North Dakota Field Station, NDSU Biological Sciences Dept. 2715, P.O. Box 6050, Fargo, ND 58108 U.S.A.

Supplementary Figure S1. Response curves from species distribution models for each predictor (defined in Table 1) and species including a) *Salvator merianae,* b) *S. rufescens,* c) *Tupinambis teguixin* and d) the three tegu species combined. Curves for each model algorithm (boosted regression trees [BRT]; generalized linear models [GLM]; multivariate adaptive regression splines [MARS]; maximum entropy [Maxent]; random forest [RF]) are paired with each background method (random and targeted).


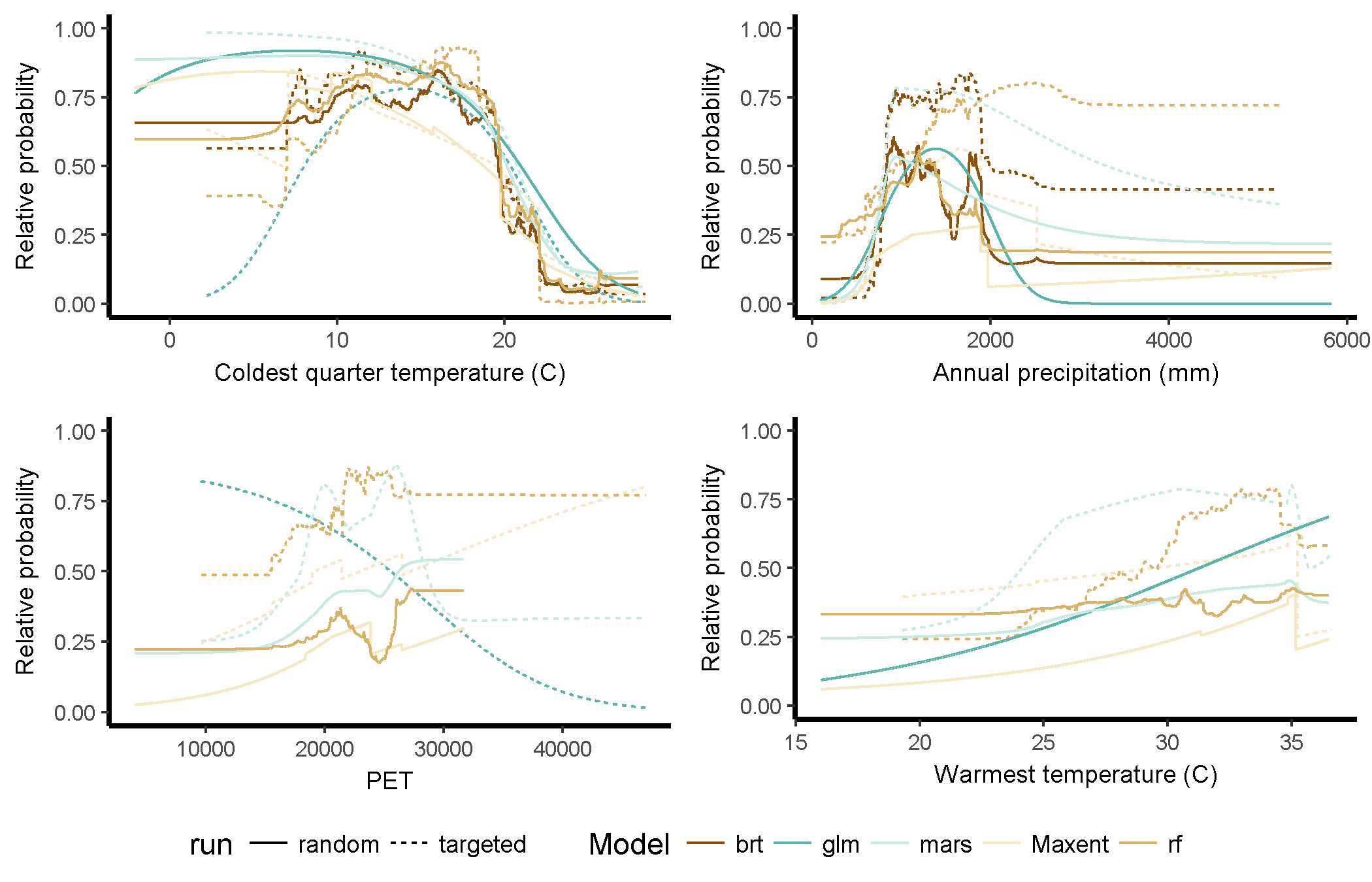


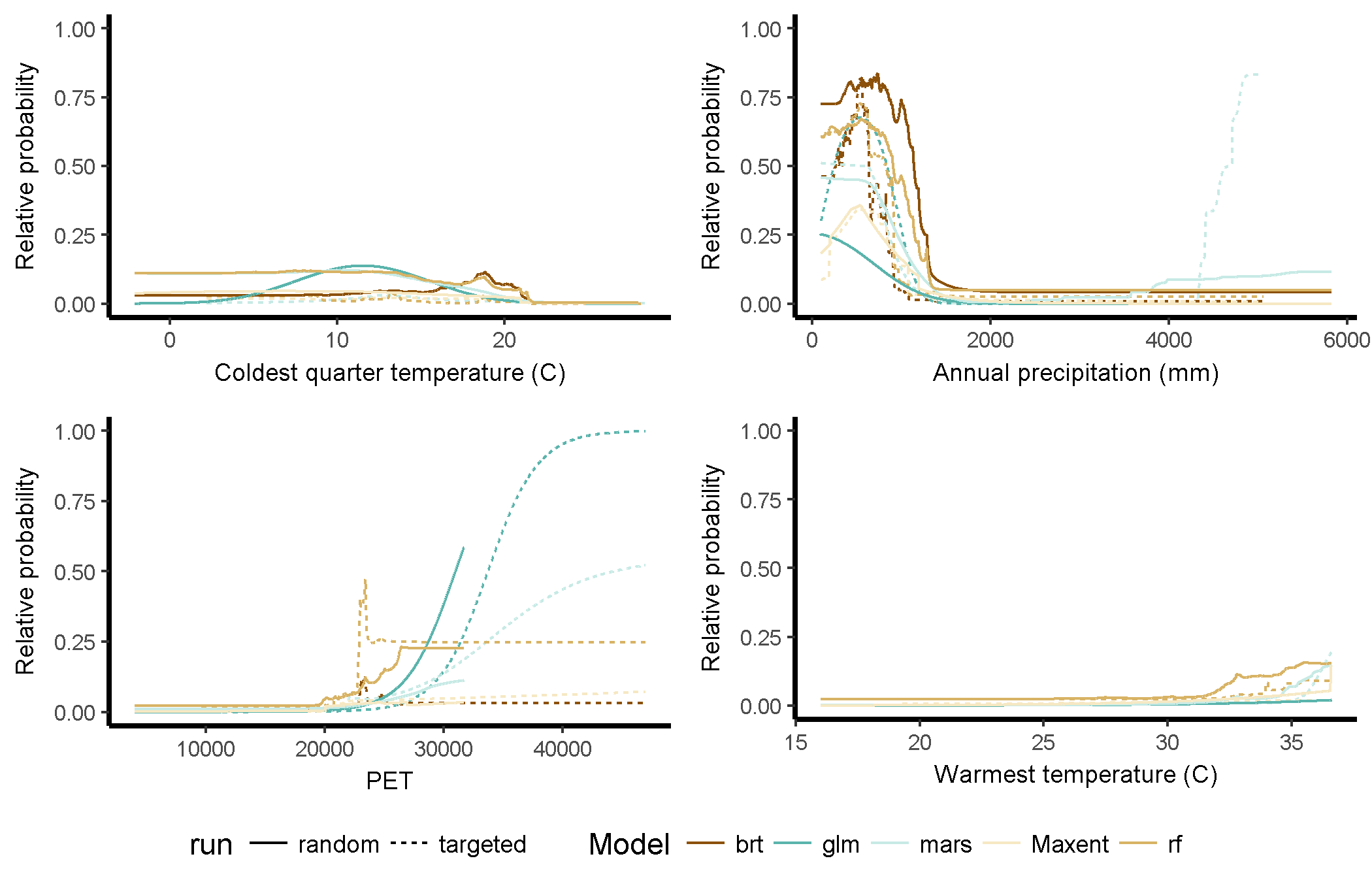


c)
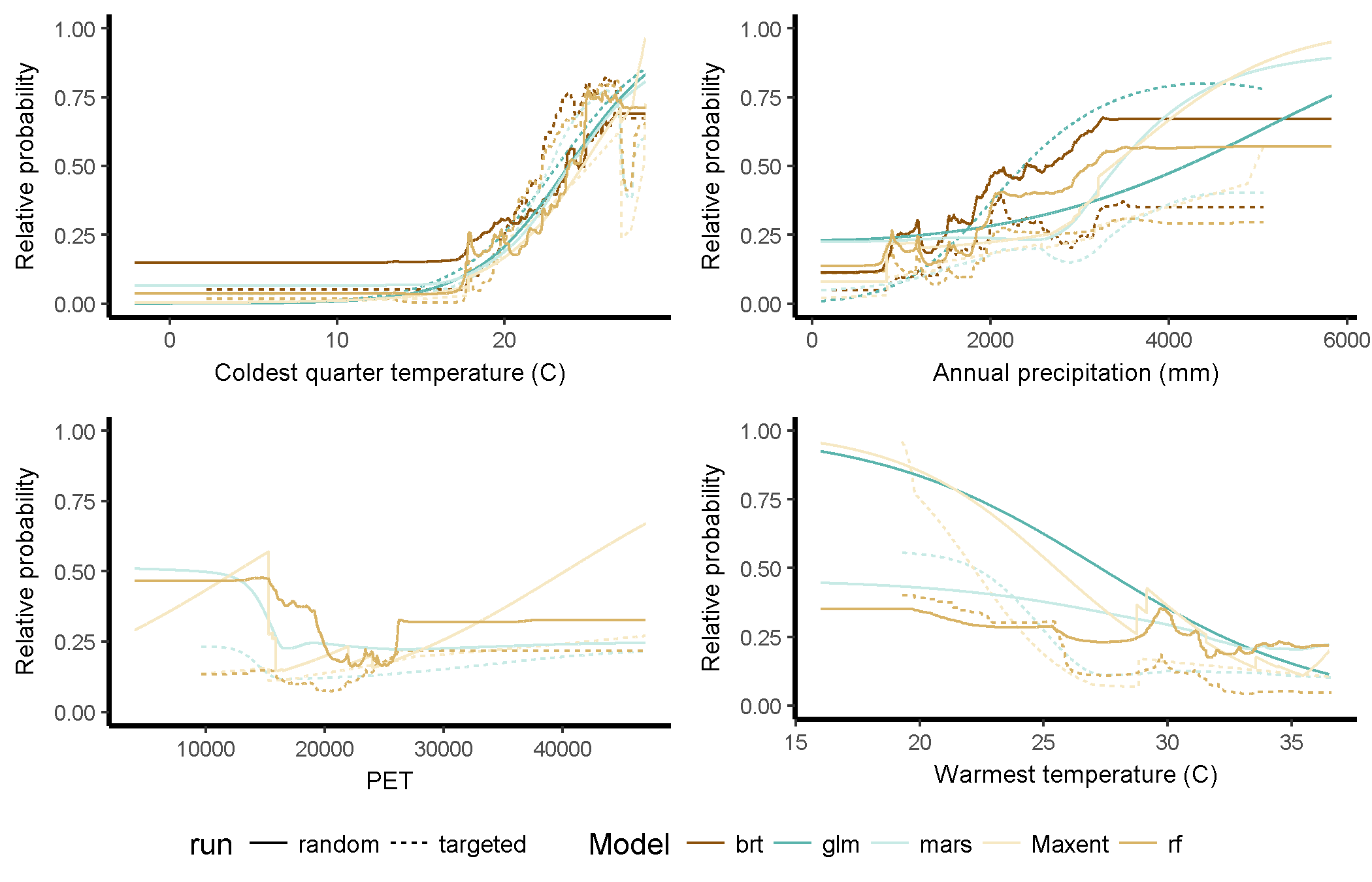


d)
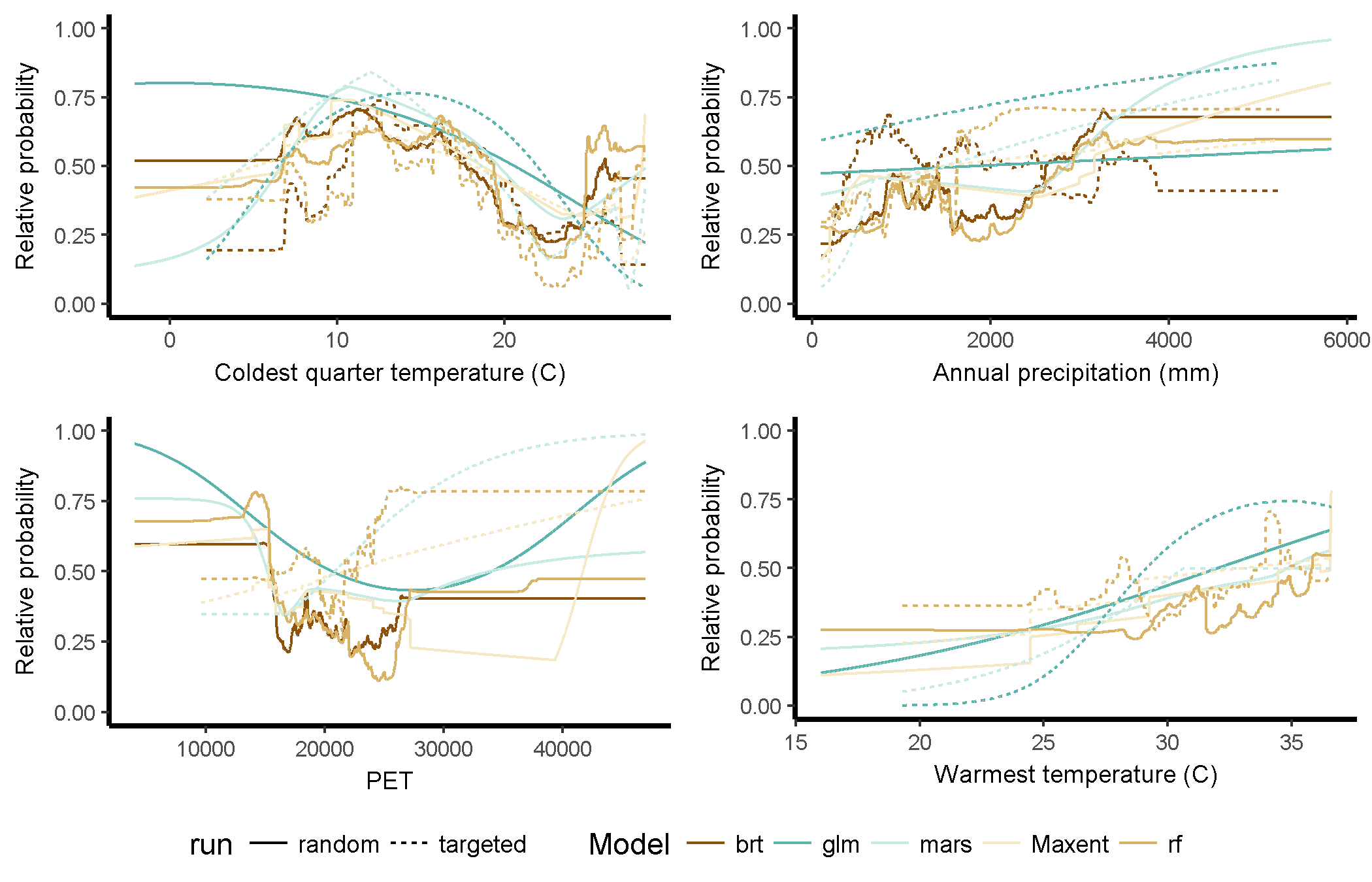


Supplementary Figure S2. Habitat suitability for a) Hawaii and the U.S. territories including b) American Samoa, c) Northern Mariana Islands, d) Guam, e) U.S. Virgin Islands, and f) Puerto Rico for models generated with either a random background approach or targeted background approach (column or row named targeted or random) for the three tegu species combined (combined) and the individual species (*Salvator merianae* [same], *S. rufescens* [saru], and *Tupinambis teguixin* [tute]; column or row named combined, same, saru, tute). Areas identified as having novel environmental conditions based on the Multivariate Environmental Similarity Surface are shown with a transparent gray layer.

a)


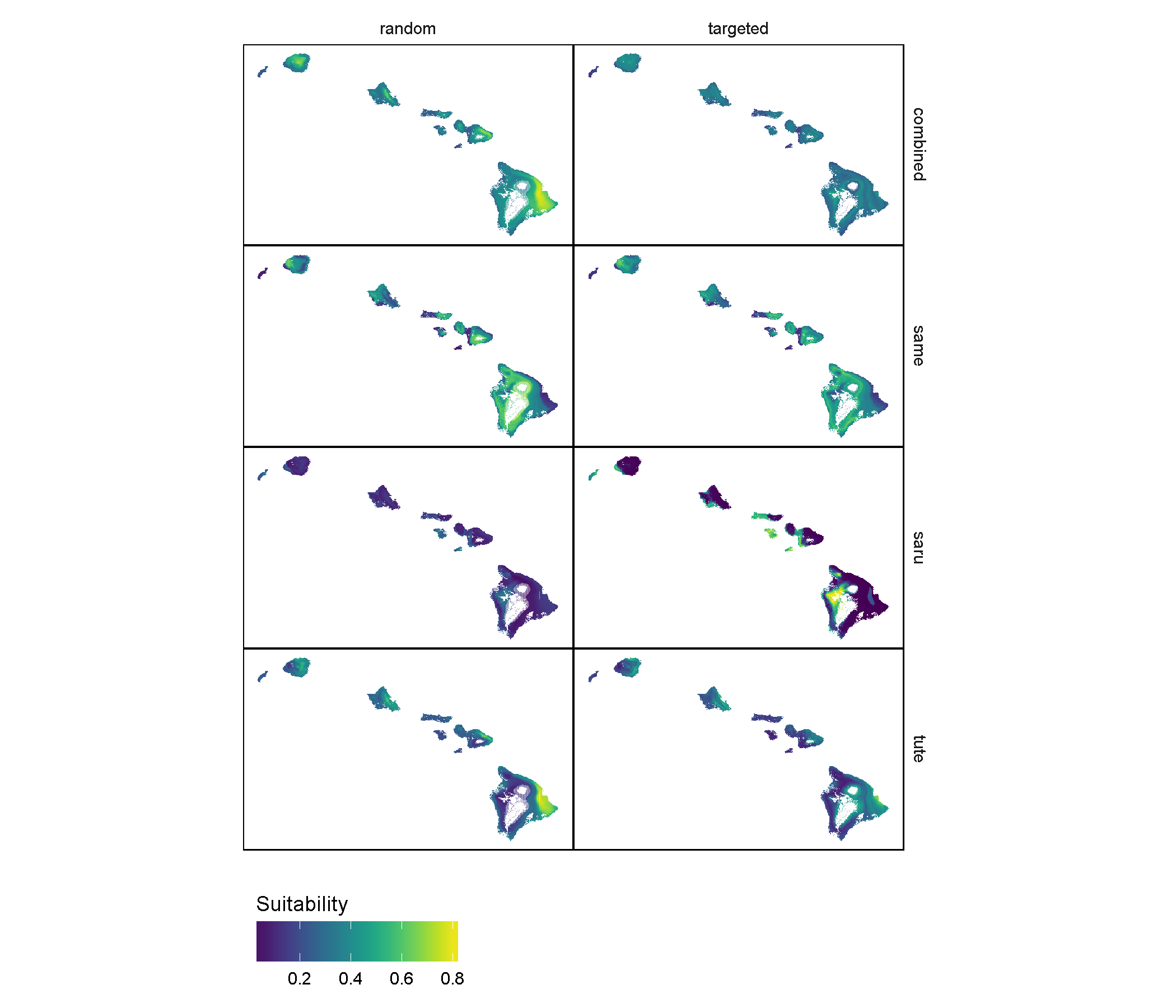


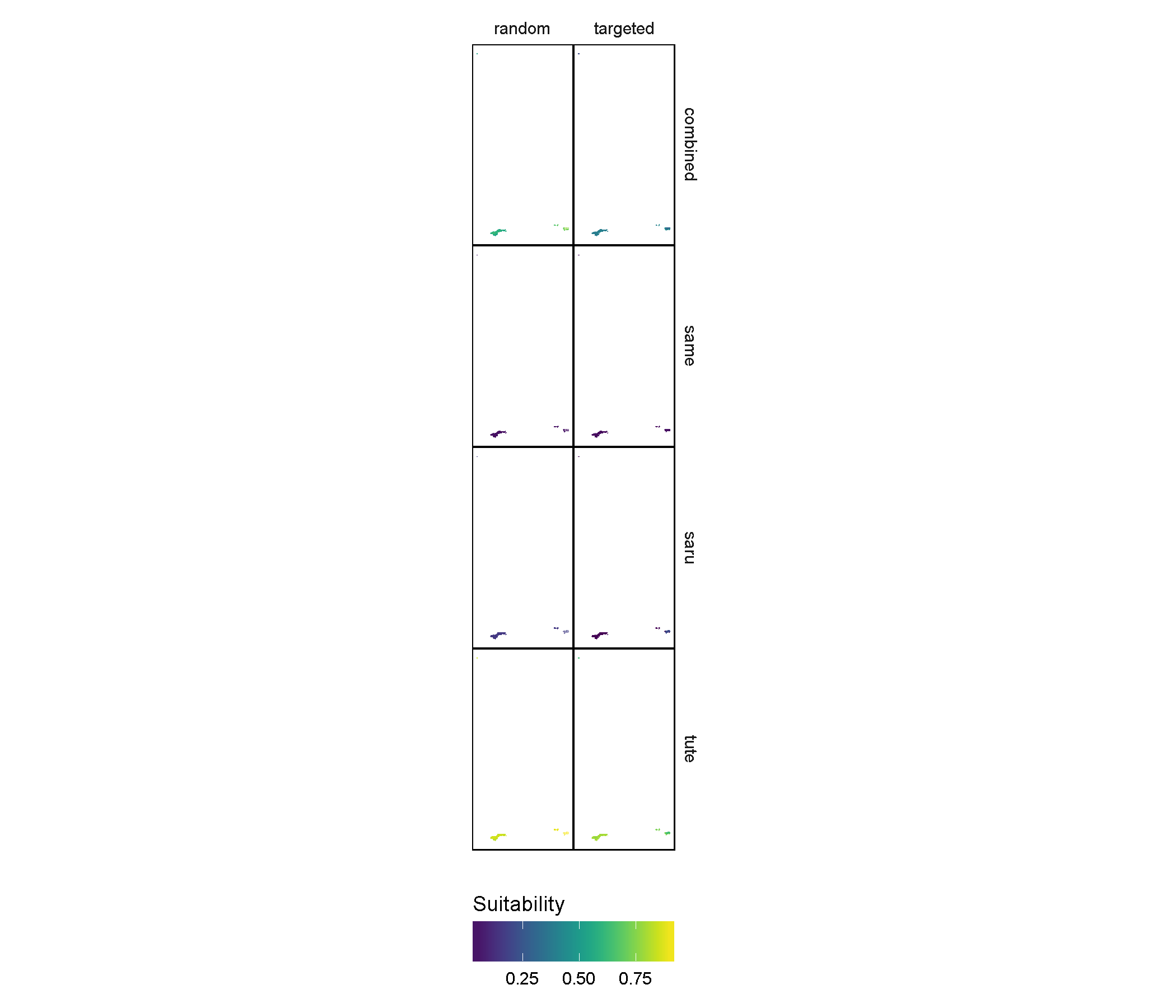
b)


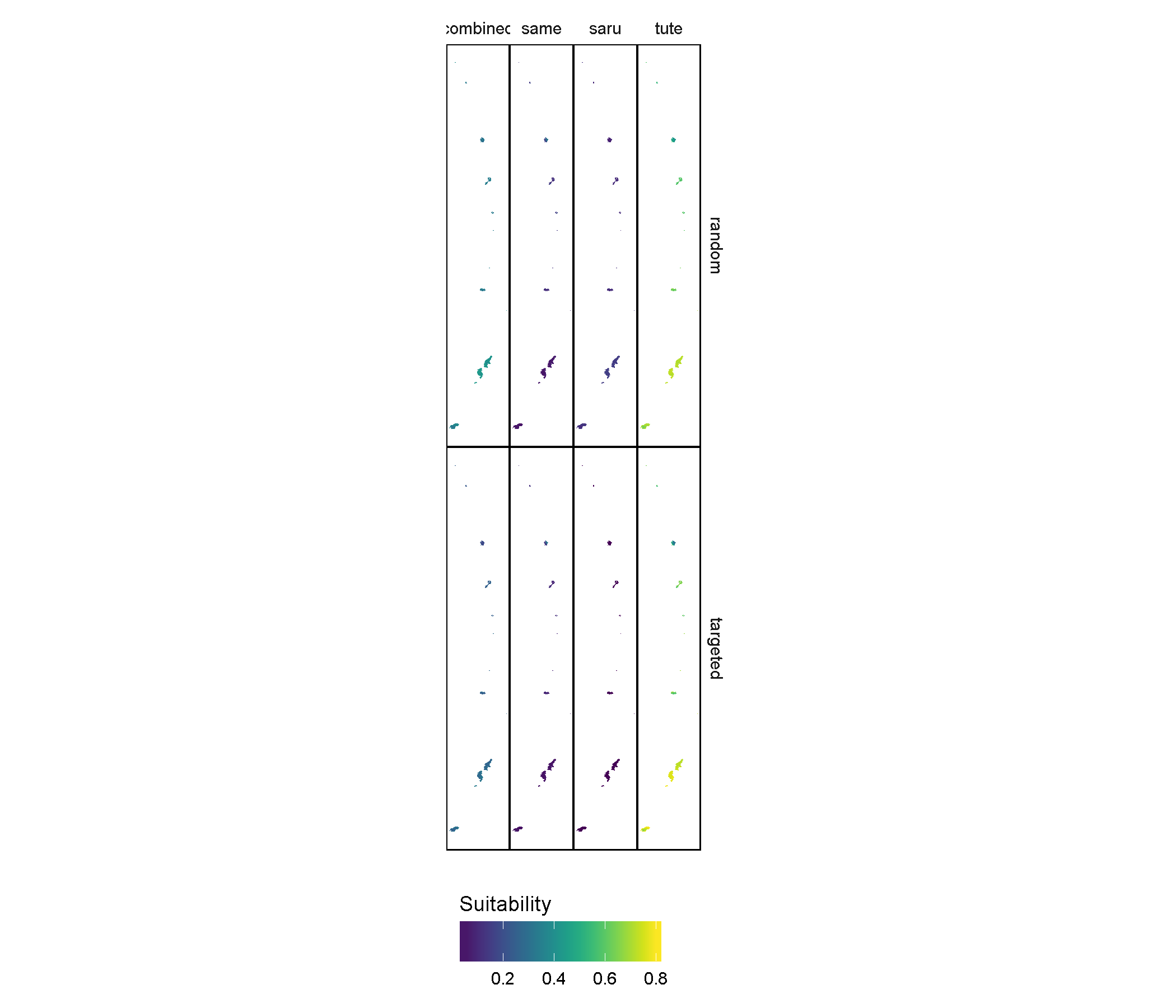
c)


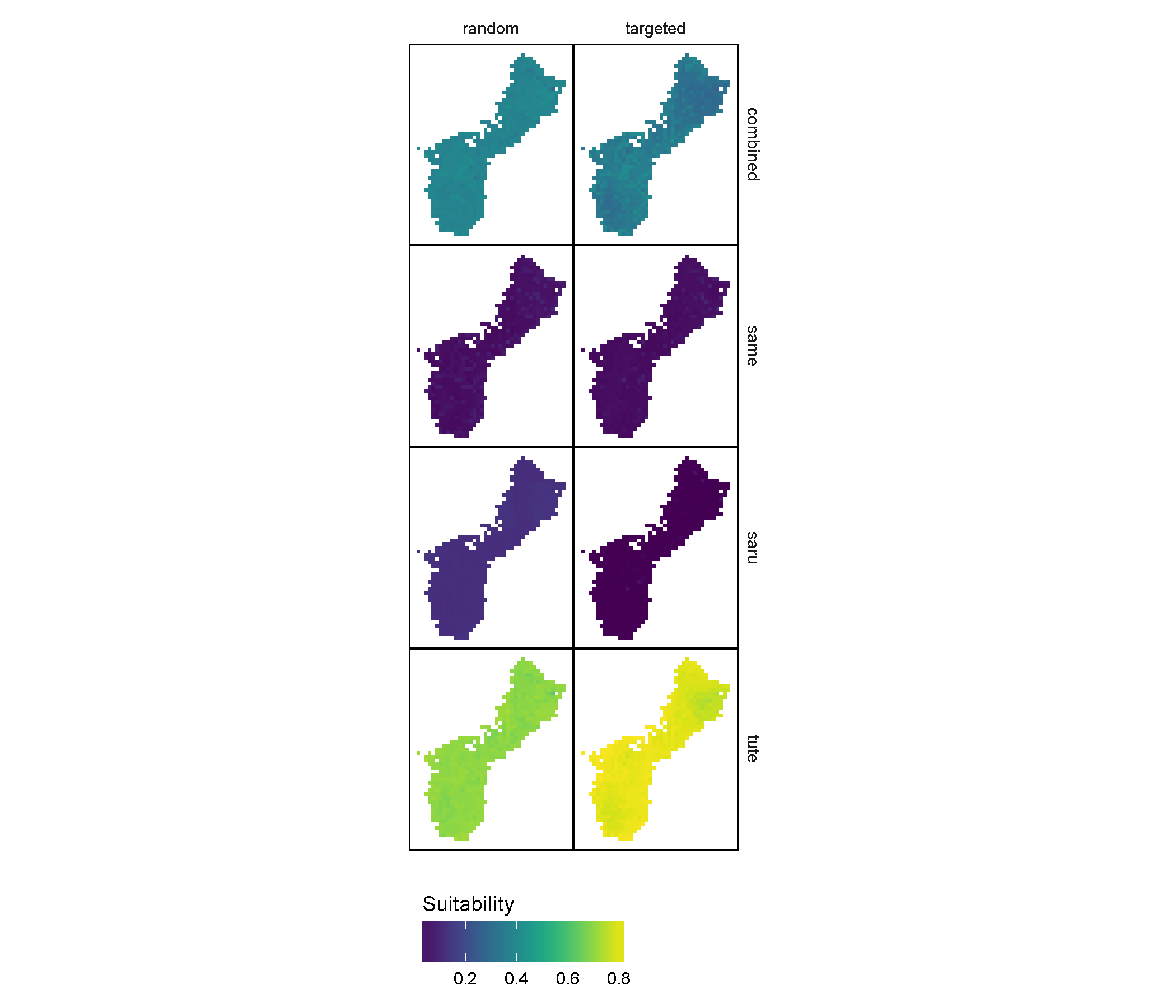
d)


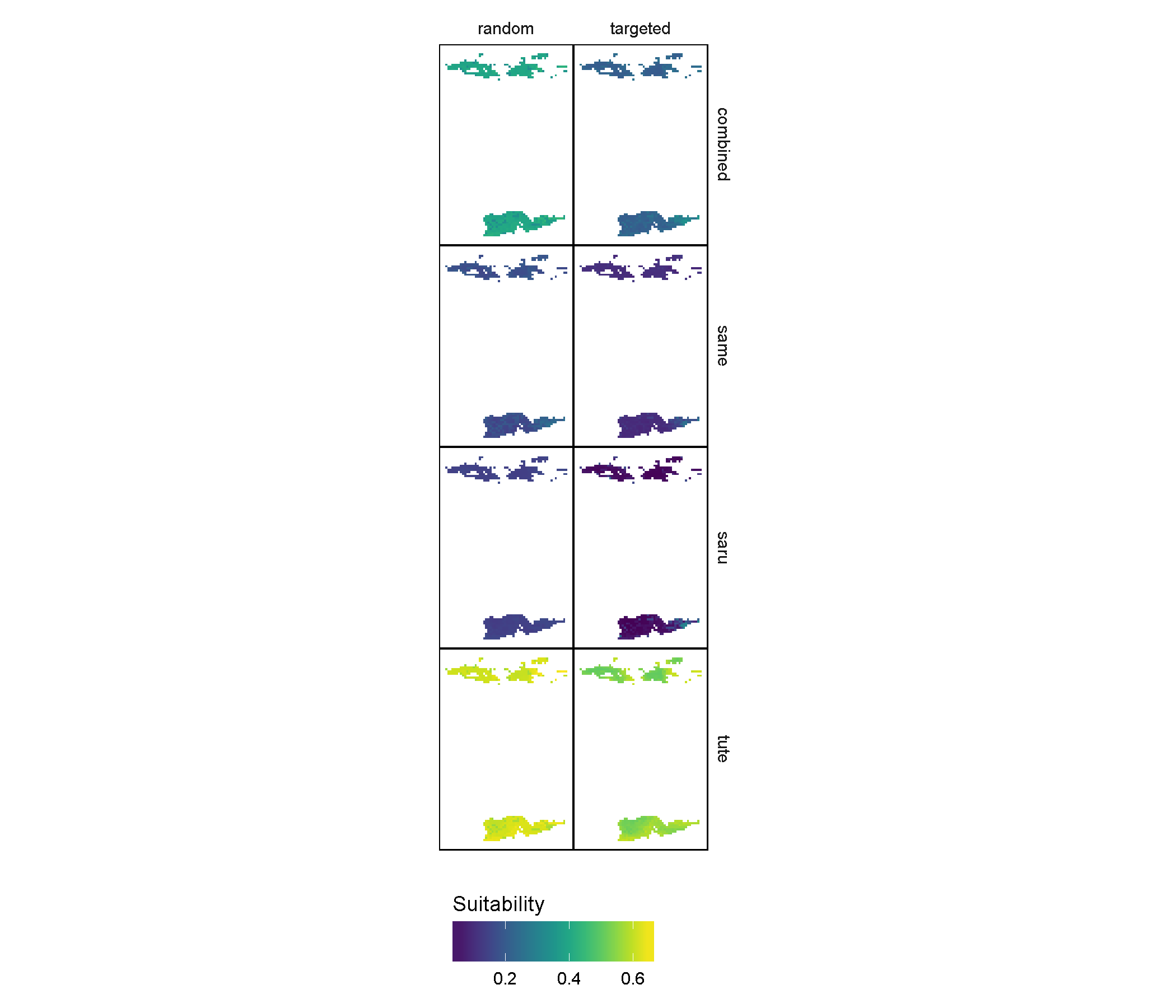
e)

f)


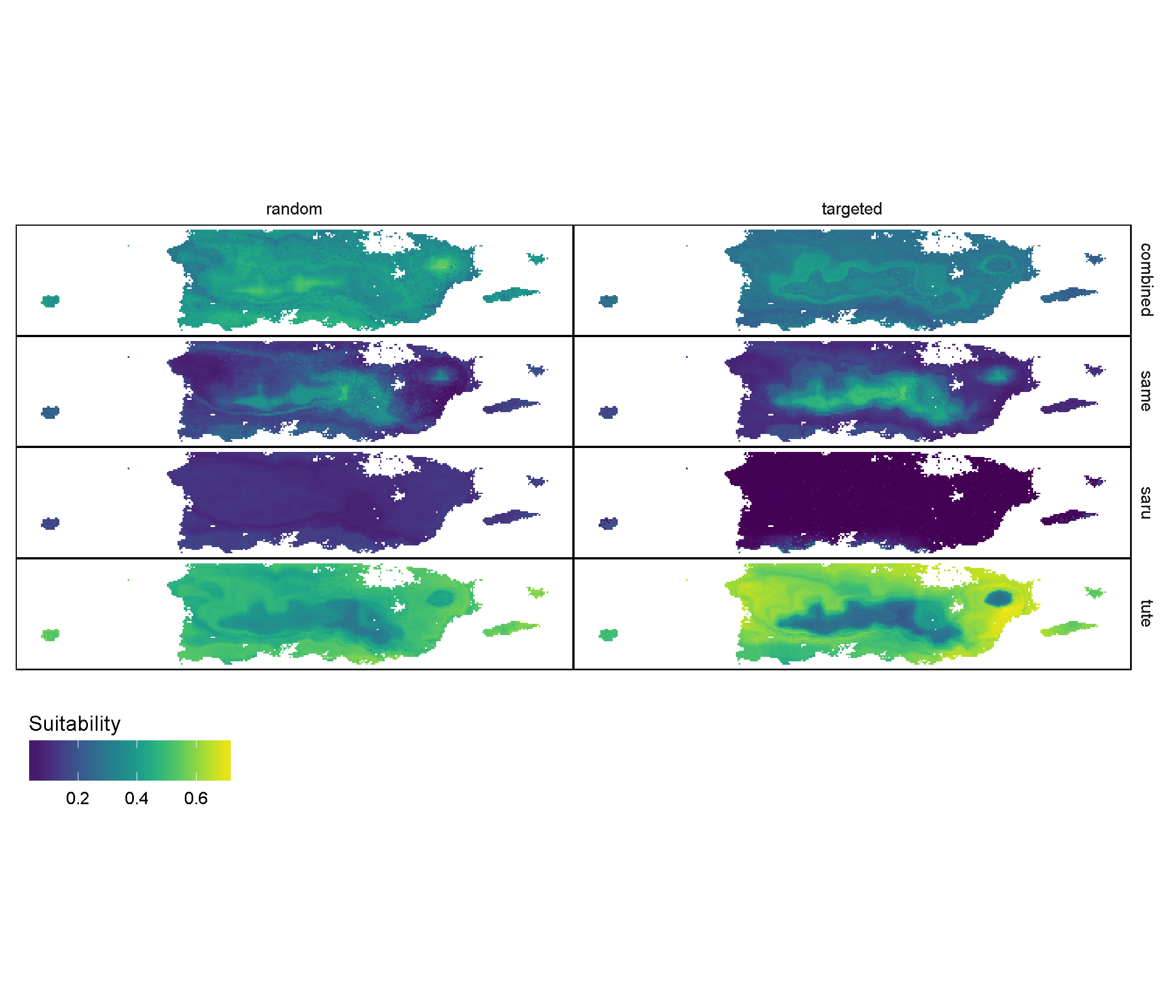

Supplement: Supplementary file 1 — Supplementary information [file 41598_2018_28468_MOESM1_ESM.doc]
